# Case report: use of pleural dialysis as an alternate means of renal replacement therapy in three cats

**DOI:** 10.3389/fvets.2024.1447629

**Published:** 2024-10-22

**Authors:** Mara E. Vernier, Meghan E. Fick, Tyler E. Johnson, Yu Ueda, Alessio Vigani

**Affiliations:** ^1^Department of Small Animal Medicine and Surgery, University of Georgia, Athens, GA, United States; ^2^Department of Clinical Science, North Carolina State University, Raleigh, NC, United States; ^3^Department of Small Animals, University of Zurich, Zurich, Switzerland

**Keywords:** dialysis, pleural, kidney, feline, azotemia

## Abstract

**Objective:**

The objective of this case series is to describe the indications, methodology, and short-term outcomes of three cats with severe azotemia managed with pleural dialysis.

**Case summary:**

Three cats were presented separately to the emergency room (ER) on referral for severe azotemia of varying etiologies. Despite aggressive medical and/or surgical management, none of the cats showed improvement in their blood urea nitrogen (BUN) or creatinine values. Renal replacement therapy was recommended, but for varying reasons, the patients were unable to undergo a traditional extracorporeal method, such as intermittent hemodialysis (IDH). Instead, pleural dialysis was performed, and all three cats showed improvement in their renal values during and after their treatment. No significant complications were documented as a result of pleural dialysis. Two of the three cats were discharged from the hospital and the third cat was humanely euthanized due to poor prognosis.

**New or unique information provided:**

Pleural dialysis is a novel therapeutic procedure that is not documented in veterinary or human literature. This method of renal replacement therapy was well-tolerated and had no reported complications. Careful case selection and risk-benefit analysis should be considered before attempting this procedure. Further studies are necessary to further define the utility of this therapeutic intervention, evaluate the incidence of complications, and determine long term outcomes following the procedure.

## 1 Introduction

Renal replacement therapy (RRT), a crucial tool in veterinary medicine, is primarily used to manage severe uremia. There are no conventional medical therapies that are as efficacious as RRT to treat acid-base, biochemical, and fluid abnormalities associated with renal disease. In the past few decades, there has been a substantial demand for RRT, such as hemodialysis. The goal of renal replacement therapies is to alter the composition of body fluids by exposing the patient's blood to a prescribed solution, the dialysate, across a semipermeable membrane. In traditional hemodialysis, the membrane utilized is a commercially produced dialyzer. In some situations, hemodialysis is unavailable, and alternative methods are necessary, underscoring the critical need for additional effective renal replacement therapies in veterinary medicine.

Peritoneal dialysis has been thoroughly described in veterinary medicine. By utilizing the unique properties of the peritoneum's semipermeable membrane to act as a filter, the use of a dialysate instilled into the peritoneum can aid in correcting water, solute, and acid-base disturbances, as well as the removal of uremic and other dialyzable toxins. Peritoneal dialysis has been described as a successful treatment strategy for acute or chronic kidney injury in small-breed dogs (1). The choice of peritoneal dialysis over other forms of renal replacement therapy is ultimately case-dependent. However, it is most commonly utilized due to cost, ease of supply accessibility, and in cases where venous access is difficult to obtain (1, 2).

This report piques interest by introducing a novel technique, pleural dialysis, for managing severe azotemia in three feline patients. The pleural membrane, histologically and anatomically similar to the peritoneal mesothelium, consists of a simple epithelial monolayer, basement membrane, and interstitium with microvasculature (2, 3). With a similar anatomic structure, the pleura can act as a filter like the peritoneum via the 3-pore theory (2). The barrier to diffusion and osmosis between the peritoneal space, and therefore the pleural space, and blood lies within the anatomy of the walls of the capillaries and the extracellular matrix within the submesothelial cell connective tissue. These barriers allow for limited movement of macromolecules, which move via large pores due to their small numbers within the peritoneum. The movement of small solutes is dictated by the number of small pores, which appear in great numbers. The passage of water occurs via ultrasmall pores that contain aquaporin I channels and are inducible in various states. This report demonstrates the use of a novel technique, pleural dialysis, for managing severe azotemia in three feline patients who had previously failed traditional medical and/or surgical management for varying causes of azotemia, sparking curiosity and potential avenues for further research and patient management.

## 2 Case summaries

### 2.1 Case 1

A 7-year-old female spayed domestic shorthair cat was presented to a tertiary referral hospital for a newly diagnosed anemia and azotemia. The patient had become progressively hyporexic over the past few months and initially presented to her referring veterinarian for anorexia and chronic weight loss. Bloodwork revealed a normocytic, normochromic anemia with a hematocrit of 18%, a moderate leukocytosis, and a marked azotemia [blood urea nitrogen (BUN) of 103 mg/dL and a creatinine of 12.5 mg/dL]. She was discharged after receiving subcutaneous fluids and an injection of cefovecin. The following day, she presented to an emergency clinic for persistent lethargy and anorexia. There, she was hospitalized overnight with intravenous fluid therapy and supportive care. A repeat complete blood count (CBC) showed persistent anemia with a packed cell volume (PCV) of 15% and total protein (TP) of 6.0 gm/dL. Serum chemistry showed mild improvement of azotemia (BUN: 108 mg/dL and creatinine: 6.4 mg/dL), but a new hyperkalemia (7.7 mmol/dL). An abdominal ultrasound showed left hydronephrosis with significant left ureteral dilation. She was then referred to the author's facility for further assessment.

On presentation, the patient was deemed stable with normal vital parameters and a normal cardiopulmonary exam. Abdominal palpation was unremarkable, except for a slightly enlarged left kidney. Bloodwork was consistent with persistent anemia (PCV: 16%) and severe azotemia (BUN: 125 mg/dL and creatinine: 7.6 mg/dL). Additionally, she was hyperphosphatemic (7.6 mg/dL) and hyperkalemic (6.4 mmol/L).

Repeat abdominal ultrasound confirmed left hydronephrosis, ureteral dilation, and ureteritis with intraluminal debris and a single ureterolith compatible with ureteral obstruction. Additionally, the right kidney was noted to have chronic degenerative changes accompanied by nephrolithiasis. Urocystoliths and mild diffuse pancreatopathy were also noted. Given the presence of new hyperkalemia, chronic degenerative changes of the right kidney, and ureteral obstruction of the left kidney, the patient underwent subcutaneous ureteral bypass (SUB) placement. Following recovery from general anesthesia, the patient was noted to develop difficulty breathing, and pulmonary infiltrates were subsequently confirmed on thoracic radiographs. Echocardiogram showed concern for mild left ventricular enlargement without concentric hypertrophy, indicating the presence of fluid overload. Her respiratory status improved following a single dose of furosemide (2 mg/kg, IV).

In the subsequent days of hospitalization, progression of azotemia (BUN: 176 mg/dL, creatinine: 10 mg/dL) was noted. A reassessment of ultrasound showed a resolution of hydronephrosis, indicating the proper function of the SUB device. The progression of her azotemia was thought to be secondary to additional acute kidney injury (AKI) of unknown etiology. As such, RRT was pursued. Dialysis catheter placement was attempted twice and unsuccessful on two separate occasions. Pleural dialysis was subsequently pursued. See Section 3 for a description of the pleural dialysis procedure.

**Figure 1:**
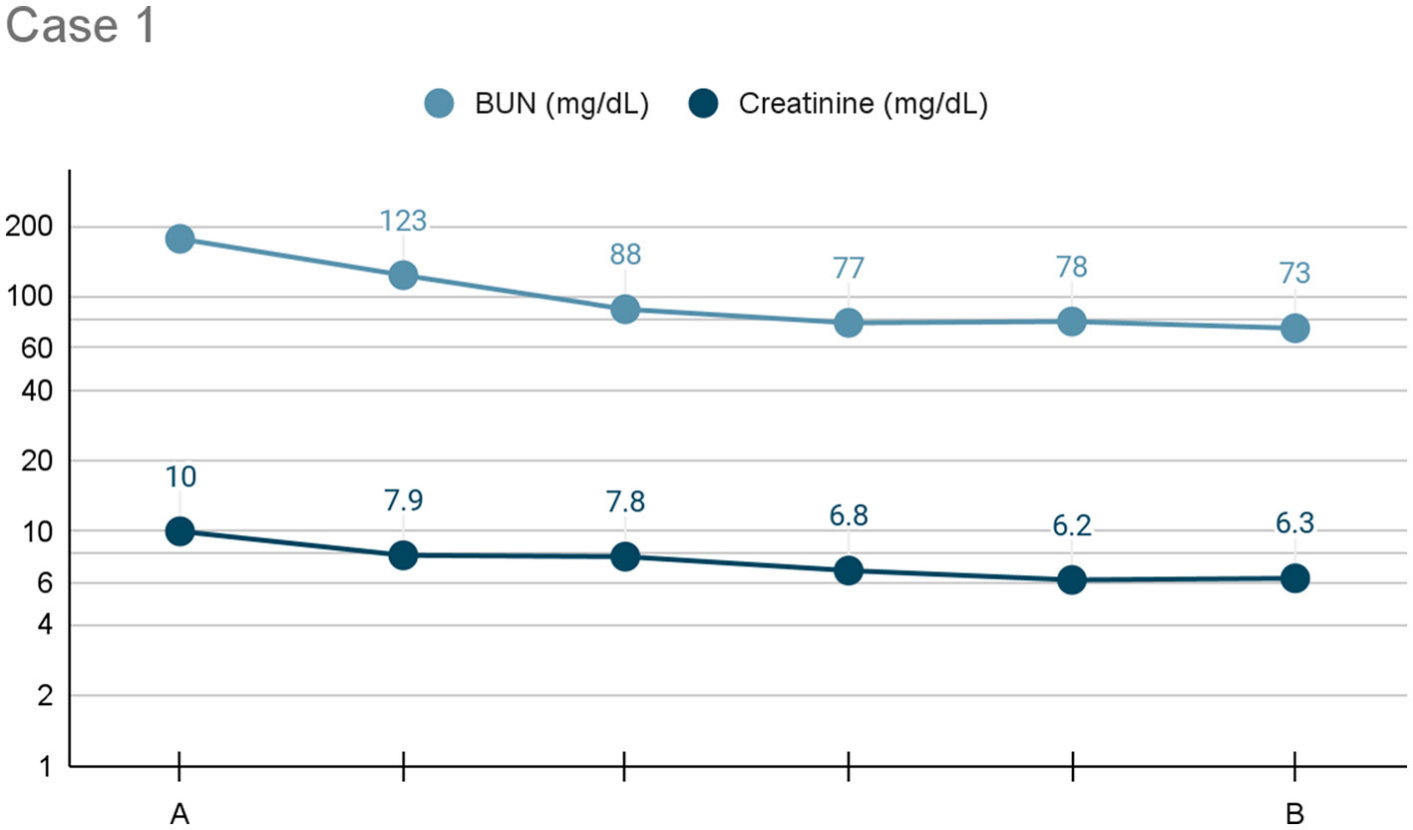
Line graph depicting the serum BUN and creatinine (*x*-axis: mg/dL) trends) from initiation **(A)** to cessation **(B)** of pleural dialysis over the course of treatment (*y*-axis: time), or 96 h, for Case 1.

Pleural dialysis was performed continuously for a total of 4 days, at which point the chest tube was no longer functional. A significant improvement in azotemia was noted, with a plateau in values (BUN: 73 mg/dL, creatinine: 6.3 mg/dL) achieved on day 4 (see Figure 1). Ideally, an additional 2–3 days of dialysis would have occurred.

The patient was discharged with traditional therapies for chronic kidney disease. A 2-week follow-up with her regular veterinarian indicated that her azotemia remained static.

### 2.2 Case 2

A 3-year-old female spayed Snowshoe cat was evaluated at the author's institution on referral following the diagnosis of severe azotemia (BUN: >180 mg/dL, creatinine: 8.9 mg/dL) and hyperphosphatemia (15.7 mg/dL) by the patient's primary veterinarian while being assessed for a 4—day history of vomiting, anorexia, and lethargy. Abdominal radiographs completed at this visit were unremarkable. A physical exam at the author's institution indicated that the patient was 6–8% dehydrated with the unremarkable cardiovascular exam. She was, however, noted to be moderately uncomfortable during abdominal palpation. Urinalysis and abdominal ultrasound were noted to be unremarkable. Urine was submitted for culture. During hospitalization for medical management, the patient was noted to have progressive azotemia, increasing body weight, and developed oliguria. RRT was indicated; however, intermittent hemodialysis was unavailable due to machine limitations. As such, pleural dialysis was pursued. See Section 3 for details related to pleural dialysis.

**Figure 2:**
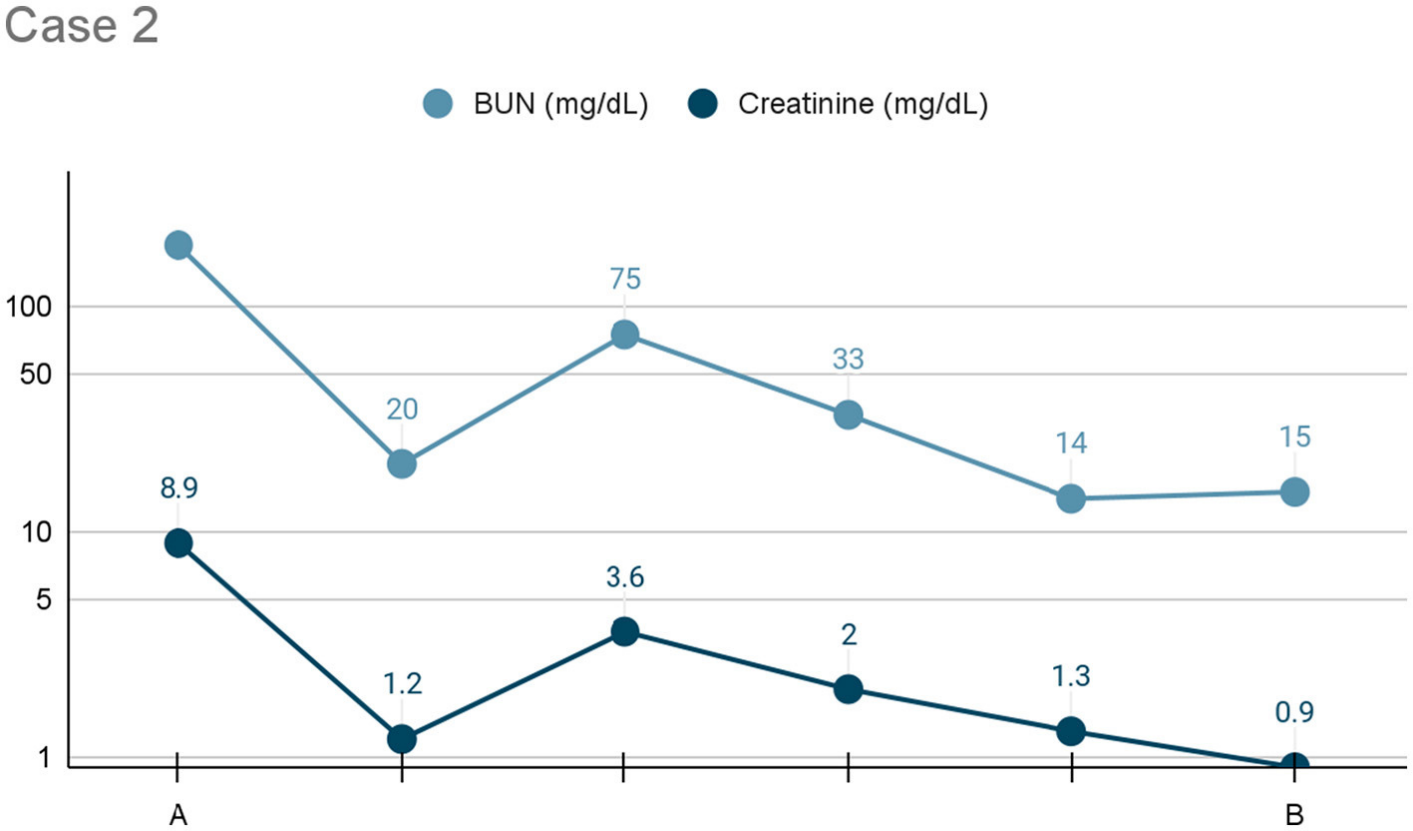
Line graph depicting the serum BUN and creatinine (*x*-axis: mg/dL) trends) from initiation **(A)** to cessation **(B)** of pleural dialysis over the course of treatment (*y*-axis: time), or 24 h, for Case 2.

After initiating pleural dialysis, the patient's renal values improved drastically after the first 24 h (see Figure 2). General trends indicated overall improvement but due to availability, different machines were utilized to trend renal values and individual variation should be interpreted with caution. Pleural dialysis was discontinued once the patient's azotemia was resolved (BUN: 20 mg/dL, creatinine: 1.2 mg/dL). After an additional 24 h, her renal values remained stable. Her urine culture was positive for Group G strep. She was discharged on Clavamox (16.6 mg/kg PO every 12 h) for treatment of the urinary tract infection and likely pyelonephritis.

### 2.3 Case 3

A 6-year-old male intact domestic shorthair cat presented for evaluation of abdominal pain and suspected mid-abdominal mass diagnosed by the primary veterinarian. Three months prior, the patient had been medically managed for urethral obstruction associated with severe azotemia (BUN: 110 mg/dL, creatinine: 8.5 mg/dL). During this hospitalization, he was noted to improve clinically; however, he maintained a persistent marked azotemia (BUN: 84 mg/dL, creatinine: 4.5 mg/dL) at the time of discharge; no follow-up occurred.

Assessment at the author's institution indicated the patient had mild discomfort on abdominal palpation with an unremarkable cardiovascular exam. Bloodwork showed evidence of marked azotemia (BUN: 228 mg/dL and creatinine: 23.3 mg/dL). Abdominal ultrasound identified moderate left hydronephrosis with mild proximal hydroureter, the right kidney was otherwise unremarkable. Given the severity of the change, it was considered that the patient had either a concurrent pyelonephritis affecting right renal function or chronic renal disease with limited function of the right kidney. Urinalysis was unremarkable for crystalluria and bacteriuria; however, the patient was empirically treated for pyelonephritis. Over the course of the next 4 days repeat abdominal ultrasound showed concern for static to mildly progressive left hydronephrosis. Azotemia remained progressive over this time (BUN: 249 mg/dL, creatinine: 28.2 mg/dL), Given patient stability it was elected to pursue pleural dialysis in order to further stabilize the patient prior to consideration of SUB placement.

**Figure 3:**
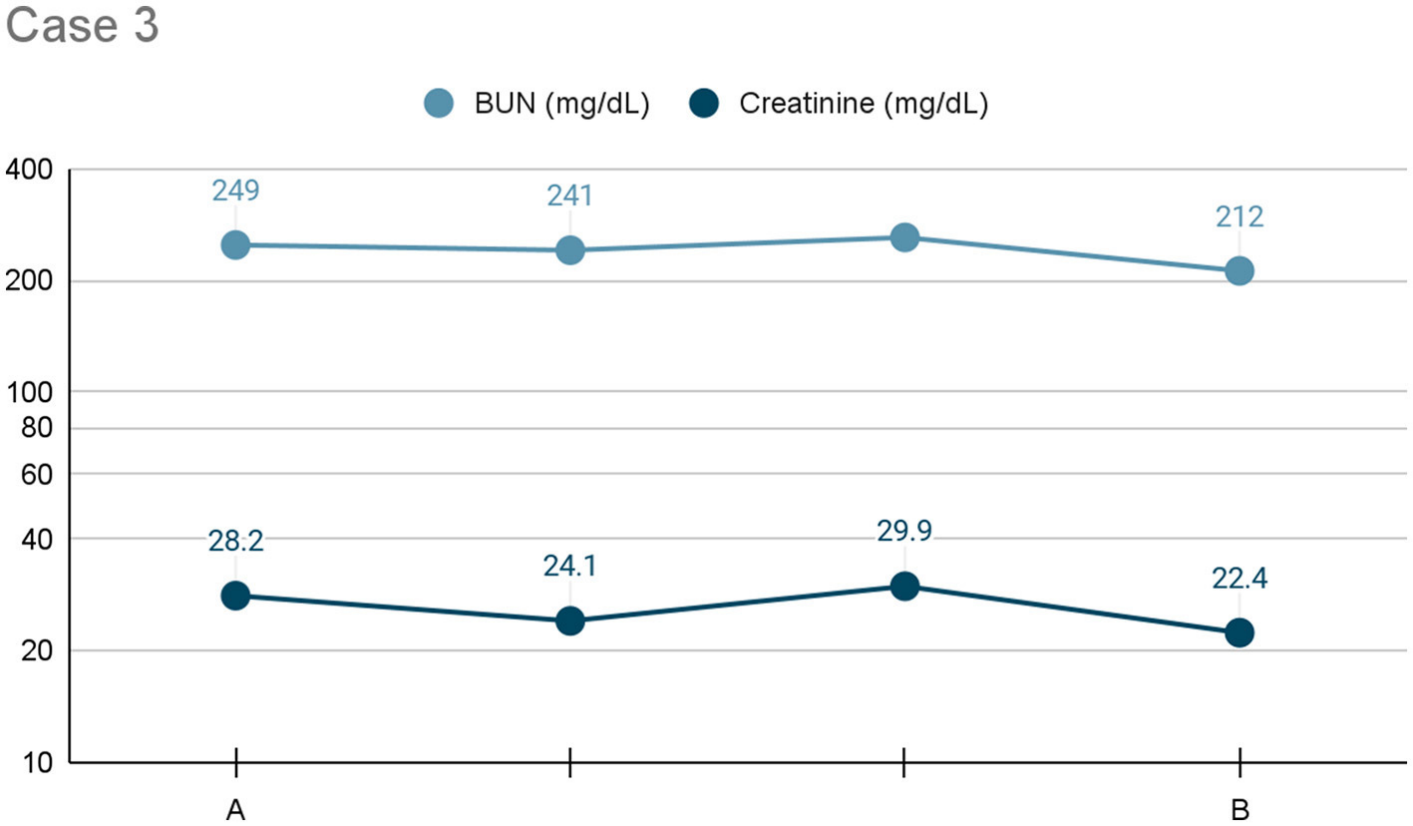
Line graph depicting the serum BUN and creatinine (*x*-axis: mg/dL) trends) from initiation **(A)** to cessation **(B)** of pleural dialysis over the course of treatment (*y*-axis: time), or 24 h, for Case 3.

The patient received pleural dialysis for ~24 h. His azotemia (BUN: 212 mg/dL, creatinine: 22.4 mg/dL) improved but remained severe (see Figure 3). Bilateral renal aspirates were obtained, and no cytologic abnormalities were identified. The patient developed hyperbilirubinemia (0.8 mg/dL) with no underlying cause identified. Considering the failure to respond to aggressive therapy and overall poor prognosis, humane euthanasia was elected. A necropsy revealed gross bilateral renomegaly, uremic lingual ulcers, and diffuse hepatic congestion. A ureterolith was identified within the left ureter, causing mild to moderate hydronephrosis. Histopathology of the right kidney revealed neutrophilic interstitial nephritis.

In contrast, enlargement of the left kidney was attributed to marked tubular hypertrophy and hyperplasia, likely occurring as a compensatory response to the atrophic chronic changes in the right kidney. Moderate to marked numbers of crystals were present in the tubules of both kidneys, although most prevalent in the left kidney. The neutrophilic interstitial pattern of injury in the right kidney was suggestive of systemic infection as opposed to ascending urogenital tract infection, but the underlying etiology was not investigated further. No changes within the thoracic cavity were noted, indicating that complications were not associated with the procedures used to perform pleural dialysis (i.e., placing chest tubes and instilling fluid into the pleural space).

## 3 Method—Pleural dialysis

All patients were adequately sedated based on clinician preference and placed in right lateral recumbency. A 7 French double-lumen central venous catheter was sterilely placed into the pleural space midway between the rib heads and costochondral junction in the 7th intercostal space and directed caudally using the modified Seldinger technique. Confirmation of correct catheter placement was achieved by thoracic ultrasound while instilling dialysate. One lumen would function as the inflow line and the other as the outflow. Dialysate was made by adding maintenance potassium chloride (0.05 mEq/kg/h) to a 5 L bag of Plasmalyte 148 with 5% dextrose (500 mL of 50% dextrose). The dialysate was hooked up to one lumen of the central venous catheter via a fluid pump. The dialysate was hooked up to one lumen of the central venous catheter via a fluid pump. A heating pad was placed around the dialysate bag to keep it warm.

Ten milliliters per kilogram of dialysate was instilled into the pleural space with an initial dwell time of 30 min, and the inflow rate was then based on the patient's urea reduction ratio as calculated by the following equation: (pre-treatment BUN – post-treatment BUN)/pre-treatment BUN. To minimize the chances of disequilibrium, a urea reduction ratio of 30% over 24 h was used, which equated to 0.25 L/kg of dialysate over 24 h.

The outflow lumen of the chest tube was also attached to fluid lines and a fluid pump to keep the outflow rate the same as the inflow rate. This fluid waste was collected in a sterile urine bag. A pressure transducer was connected to the outflow line to measure and maintain the negative pressure in the pleural space (−2 and −5 mmHg). A 3-way stopcock was attached to a sterile syringe, and another fluid line and bag of PlasmaLyte 148 in order to have a way to troubleshoot the catheter if there was any malfunction or clogging. This entire circuit was left untouched throughout the dialysis session and was maintained as a closed, sterile circuit.

The amount of time pleural dialysis was performed was dependent on the patient's clinical response and the development of complications, such as catheter malfunction, that led to premature cessation despite continued clinical improvement. All three patients experienced intermittent kinking or clogging of the catheter which required minor adjustments or flushing. The catheter in the first case ultimately clogged to the point where it was no longer functional. There was no significant dialysate loss or fluid gain in any of the three cases, with pleural fluid recovery between 92 and 99%. Ideally, dialysis was continued until azotemia was corrected or kidney values plateaued, indicating the presence of confounding chronic changes.

## 4 Discussion

This case series demonstrates the use of pleural dialysis to treat refractory azotemia in three cases. Given that all three patients were either unable to undergo intermittent dialysis, had intra-abdominal disease, or had recently undergone abdominal surgery, the pleural membrane over the peritoneal membrane was utilized to stabilize each patient further. Traditionally, hemodialysis in any form is considered for patients with severe acute kidney disease, which has caused major electrolyte and acid-base derangement, led to oligo-anuric status, and or significant volume overload. Additionally, hemodialysis can be considered for any severely azotemic patient to optimize their care while undergoing therapy for the underlying etiology of AKI (5).

The principles of diffusion and convection across a membrane are utilized in hemodialysis to remove uremic toxins (4, 5). Utilizing the principle that substances will move down their concentration gradient across semipermeable membranes from the patient's blood into the dialysate, the pleural membrane provided a semipermeable membrane for which diffusion could occur (6). When determining the suitability of a membrane, the thickness, effective surface area, number, size, and shape of its pores or diffusion channels determine the effectiveness of a membrane (5). The pleural membrane is similar to the peritoneal membrane, so it was considered an ideal alternative in the abovementioned cases.

The main determinants of whether a solute will pass via diffusion are molecular weight, solute distribution volume, and solute protein binding level. It is understood that water, along with low-molecular-weight solutes such as BUN and creatinine, each having molecular weights of 18, 60, and 113 Daltons, respectively, will readily diffuse through small pores within a membrane. However, larger solutes, such as plasma proteins like albumin with a molecular weight of 60,000 Daltons, are restricted from diffusion by pore size (7). Small solutes diffuse faster than larger solutes, so the plasma concentration will decrease faster than larger solutes throughout dialysis (8). Which was a determining factor when deciding on each patient's dialysis prescription. Because all three had similarly severe azotemia, the urea reduction ratio of 30% over 24 h was utilized for all three cases.

Peritoneal dialysis involves instilling a prescription-based dialysate into the peritoneal cavity. As explained above, diffusion allows water, uremic toxins, electrolytes, and other small molecules to equilibrate across the peritoneal membrane. Since efficient diffusion is the main mechanism responsible for solute transport in peritoneal dialysis, the dialysate is formulated based on patient needs and understanding uremic toxin diffusion kinetics. Dialysates are constructed from purified water, concentrated electrolytes, and dextrose solutions. In the above cases, commercially available PlasmaLyte 148 was utilized as the base dialysate fluid, while dextrose and potassium were added to maintain electrolyte stability and promote diffusion by increasing fluid osmolality.

The three-pore model, which details the pore sizes throughout the peritoneum, also helps explain the methodology behind using cavitary membranes as dialysis membranes. The close association between the capillaries supplying blood to the cavitary membrane is the limiting factor in solute and water transport (9). However, the membrane pore size on the membrane side of the association dictates which molecules can move freely in large numbers vs. limited numbers. Limited numbers of large pores exist, which prevents the transport of macromolecules like proteins. However, a small amount of these substances are lost via convection. Small pores are present in larger quantities and account for the movement of permeable substances such as BUN, creatinine, electrolytes, and water to some degree. Ultrafiltration occurs via ultrasmall, transcellular pores (10).

The cases presented in this paper highlight the feasibility of pleural dialysis for patients with intraabdominal pathology precluding peritoneal dialysis or in situations where machine-mediated intermittent hemodialysis is not readily available. These cases highlight a theoretically similar efficacy to peritoneal dialysis and underscore similar monitoring techniques for determining efficacy compared to intermittent hemodialysis. The first case in the series provided insight into the use of pleural dialysis in this report as none of the attending clinicians had performed such a procedure previously, and veterinary literature is not available on this dialysis method, which allowed for its utilization in subsequent cases.

Additionally, this report highlights a successful alternative treatment modality for patients who present with various etiologies of AKI that are not responsive to traditional medical or surgical management. Because this dialysis method was completed in feline patients, it could be considered when faced with small patients who could become hemodynamically unstable once the extracorporeal blood volume needed to undergo intermittent hemodialysis is utilized or if blood resources for machine priming are limited. The patients presented in this case series ranged from mild improvement to complete resolution of their azotemia. Additional prospective research is needed to understand the benefit of pleural dialysis over peritoneal dialysis in a similar patient population. This report demonstrated limited complications associated with pleural dialysis. The most common complication was obstruction of the inflow/outflow tubing. In summary, pleural dialysis is a feasible, safe, and efficacious dialysis modality that could be easily utilized in small veterinary patients.

## Data Availability

The original contributions presented in the study are included in the article/supplementary material, further inquiries can be directed to the corresponding author.
